# Anthropometric prediction models of body composition in 3 to 24month old infants: a multicenter international study

**DOI:** 10.1038/s41430-024-01501-0

**Published:** 2024-09-20

**Authors:** Vithanage Pujitha Wickramasinghe, Shabina Ariff, Shane A. Norris, Ina S. Santos, Rebecca Kuriyan, Lukhanyo H. Nyati, Jithin Sam Varghese, Alexia J. Murphy-Alford, Nishani Lucas, Caroline Costa, Kiran D. K. Ahuja, S. Jayasinghe, Anura V. Kurpad, Andrew P. Hills, Multi-center Infant Body Composition Reference Study (MIBCRS), Shabina Ariff, Shane A. Norris, Ina S. Santos, Rebecca Kuriyan, Nishani Lucas, Kiran D. K. Ahuja, Anura V. Kurpad, Andrew P. Hills, V. Pujitha Wickramasinghe, Alexia Murphy-Alford, Lukhanyo Nyati, Caroline S. Costa, Tanvir Ahmad, Jeff M. Beckett, Renata M. Bielemann, Nuala M. Byrne, Laila Charania, Michele Peresh Christian, Priscilla J. Divya, Anne Hanley, Manoja P. Herath, Leila I. Cheikh Ismail, Sisitha Jayasinghe, Pulani Lanerolle, Cornelia Loechl, Najat Mokhtar, Upul Senerath, Christine Slater, Sajid Soofi, Steven J. Street, Neiva C. J. Valle, Ayesha Yameen

**Affiliations:** 1 University of Colombo, Colombo, Sri Lanka; 2 The Aga Khan University, Karachi, Pakistan; 3 University of the Witwatersrand, Johannesburg, South Africa; 4 Federal University of Pelotas, Pelotas, Brazil; 5 St John’s Research Institute, Bengaluru, India; 6 Hubert Department of Global Health, Rollins School of Public Health, Emory University, Atlanta, USA; 7 International Atomic Energy Agency, Vienna, Austria; 8 University of Tasmania, Hobart, Australia; 9 Life Science Group, Isotope Application Division, Islamabad, Pakistan; 10 Institute of Nuclear Science and Technology (PINSTECH), Nilore, Islamabad Pakistan; 11 Department of Clinical Nutrition and Dietetics, College of Health Sciences, University of Sharjah, Sharjah, United Arab Emirates; 12 Nuffield Department of Women’s & Reproductive Health, University of Oxford, Oxford, UK

**Keywords:** Paediatrics, Nutrition

## Abstract

**Background:**

Accurate assessment of body composition during infancy is an important marker of early growth. This study aimed to develop anthropometric models to predict body composition in 3–24-month-old infants from diverse socioeconomic settings and ethnic groups.

**Methods:**

An observational, longitudinal, prospective, multi-country study of infants from 3 to 24 months with body composition assessed at three monthly intervals using deuterium dilution (DD) and anthropometry. Linear mixed modelling was utilized to generate sex-specific fat mass (FM) and fat-free mass (FFM) prediction equations, using length(m), weight-for-length (kg/m), triceps and subscapular skinfolds and South Asian ethnicity as variables. The study sample consisted of 1896 (942 measurements from 310 girls) training data sets, 941 (441 measurements from 154 girls) validation data sets of 3–24 months from Brazil, Pakistan, South Africa and Sri Lanka. The external validation group (test) comprised 349 measurements from 250 (185 from 124 girls) infants 3–6 months of age from South Africa, Australia and India.

**Results:**

Sex-specific equations for three age categories (3–9 months; 10–18 months; 19–24 months) were developed, validated on same population and externally validated. Root mean squared error (RMSE) was similar between training, validation and test data for assessment of FM and FFM in boys and in girls. RMSPE and mean absolute percentage error (MAPE) were higher in validation compared to test data for predicting FM, however, in the assessment of FFM, both measures were lower in validation data. RMSE for test data from South Africa (M/F−0.46/0.45 kg) showed good agreement with validation data for assessment of FFM compared to Australia (M/F−0.51/0.33 kg) and India(M/F−0.77/0.80 kg).

**Conclusions:**

Anthropometry-based FFM prediction equations provide acceptable results. Assessments based on equations developed on similar populations are more applicable than those developed from a different population.

## Introduction

Physical growth is one of the cardinal features of the developing child. The primary goal of all who care for children is that every child will be provided the opportunity to achieve their maximal growth and developmental potential. The early years of post-natal life are foundational for child and adult health, with nutritional status often associated with increased risk of non-communicable risk factors later in life [1–3]. Many factors influence nutritional status during early life including birth weight [4] feeding practices [4, 5], nutrient composition of the diet [6], genetics [7] and environmental factors [8, 9].

Anthropometry is typically used for assessment and results compared with standards or references to determine appropriateness. However, many anthropometric measures are problematic. Firstly, weight-for-length (WFL), and the BMI-for-age are commonly used indices to predict adiposity, but do not distinguish between fat mass (FM) and fat-free mass (FFM). Secondly, anthropometric cut-offs for these measures have been determined based on population distribution of the parameter rather than on biological or functional relevance. Thirdly, references have commonly been based on selected populations and/or populations of high-income countries resulting in inaccuracies in determining nutritional status in certain ethnic groups, most commonly, an underestimation of overnutrition and an over-estimation of undernutrition [10]. This is especially the case in populations with low birth weights who are relatively short and have low weight-for-age.

While it is understood that body composition, rather than anthropometry, is a better indicator of an infant’s nutritional status, body composition assessments may not be suitable for clinic settings or large scale epidemiological studies. Most anthropometry-based approaches are relatively easy to use, quick to produce results, inexpensive, portable and involve minimum discomfort to the child. It is therefore important to identify the best anthropometric predictive measures to reflect body composition. A number of anthropometry-based prediction equations have been developed to assess body composition in infancy [11, 12] and it has been shown that combining anthropometric measures [11, 13–15], gestational age [16], sex [17, 18], and ethnicity [9] improves predictability. However, many equations are not generalizable to populations other than those they were developed in and equations developed in multi-ethnic populations are scarce. This study aims to develop anthropometry-based prediction equations for body composition during the first two years of life in infants from diverse socioeconomic settings and ethnic groups.

## Materials and methods

### Study design and recruitment

Data are from the Multi-center Infant Body Composition Reference Study (MIBCRS), a longitudinal, prospective, multinational study, that followed infants from birth to 24 months in lower-middle (India, Pakistan and Sri Lanka), upper-middle (Brazil and South Africa) and high-income (Australia) countries and details of the recruitment process is described elsewhere [4, 19–21]. Each participating country conducted the study adhering to International Ethical Guidelines for Biomedical Research Involving Human Subjects and obtaining approval from their respective review committee. Informed written consent was obtained from the enrolled mothers and data were collected from 2013 to 2019. The main cohort comprised of 3–24 month data from Brazil, Pakistan, South Africa, and Sri Lanka from 708 mother infant pairs to assess body composition used for the development of equations (training data) and validation of the developed equations (validation data). An independent cohort comprised of 250 infants (3–6 months) from Australia, India and South Africa was used for external validation of the developed equations (test data). During follow-up, children were fed according to Infant and Young Child Feeding (IYCF) guidelines.

The sample size for study sites was calculated to have a power of 90% to detect FM and FFM for boys and girls less than one standard deviation away from a reference study, that found a mean FM of 3.10 ± 0.5 kg and 3.05 ± 0.46 kg, and mean FFM of 9.13 ± 1.06 kg and 8.99 ± 1.1 kg for boys and girls, respectively [22].

### Body composition assessed using the Deuterium Dilution (DD) technique

DD was utilized to calculate FM and FFM of infants at 3, 6, 12, 18, and 24 months of age in the development and validation group, and at 6 months of age in the test group. Details of the technique are provided elsewhere [23].

### Anthropometry

Anthropometric data for this analysis were used from the visits at 3, 6, 9, 12, 18 and 24 months in the development and validation group and from the 6 month visit in the test group. Standardized protocols for anthropometry were developed based on the WHO Multicentre Growth Reference Study (WHO-MGRS) protocol [24].

Infant weight was measured naked, using a paediatric electronic scale (Seca 376; Hamburg, Germany) and length using a Harpenden infantometer (300–1100 mm, accurate to 1 mm; Holtain Ltd, Crymych, Wales, UK) in all countries except for India and Sri Lanka (Seca 417; Hamburg, Germany). A detailed protocol has been published [23].

Triceps skinfold thickness (TSFT) and subscapular skinfold thickness (SSFT) were measured using a Holtain Tanner skinfold calliper to the nearest 0.2 mm on left arm (Holtain Ltd, Crymych Wales, UK). Each skinfold thickness was read after 2 s, consistent with the WHO-MGRS methodology with MAD of 2 mm [24]. Mid upper arm and head circumference were measured with a non stretchable flexible tape to the nearest 1 mm and MAD was 5 mm (Seca 212; Hamburg, Germany).

### Quality control in data collection

Anthropometry protocol training was undertaken in Johannesburg, South Africa and subsequently, anthropometry standardization sessions were undertaken locally at three monthly intervals. Intra- and inter-observer technical errors of measurements were calculated and compared to the measurements obtained by the anthropometry supervisor (gold standard). A training workshop on the DD technique was held at St John’s Research Institute, Bangalore, India. The IAEA organized an inter-laboratory comparison for the analysis of deuterium enrichment among the laboratories responsible for analyzing samples.

### Statistical analysis

Data were collated and captured on the REDCap system [25], hosted at the University of the Witwatersrand, Johannesburg.

### Sample split for model development

Participants from the 3 to 24-month cohort were split randomly into training (two-thirds) and validation (one-third) groups. The training data consisted of 942 sets of anthropometric and body composition measurements (collected from 310 girls) and 954 sets of measurements (collected from 340 boys). Observations corresponding to the 24-month visit were included only up to 26 months of age. The validation data set consisted of 441 sets of measurements (collected from 154 girls) and 500 sets of measurements (collected from 170 boys) from the same four country cohorts. Test data for external validation of the fitted model consisted of participants from three birth cohorts in Australia (21 girls, 30 boys with one set of measurements each at 6 months), India (44 girls, 46 boys, with one set of measurements each at 6 months) and South Africa (120 sets of observations, collected from 59 girls and 88 sets of measurements collected from 50 boys, who provided data during at least one visit between 3 to 6 months). Additional information on the sample selection is provided in Supplementary Fig. 1. We describe the characteristics of our training, validation, and test data separately by sex using median and range (Table 1 and 2).

**Table 1:** Comparison of age, anthropometric and body composition measurements between the training and validation samples between 3 and 24 mo of age for each sex, pooled and for each country.

	Pooled		Brazil		Pakistan		South Africa		Sri Lanka	
	Training	Validation	Training	Validation	Training	Validation	Training	Validation	Training	Validation
Boys										
Number of observations	954	500	306	154	192	109	317	154	139	83
Age (mo)	9.1 (2.3, 25.7)	9.2 (2.4, 25.7)	10.4 (2.7, 25.5)	9.1 (2.7, 25.4)	9.1 (2.9, 25.7)	9.1 (2.9, 25.7)	11.6 (2.3, 24.8)	11.7 (2.4, 24.3)	7.4 (2.7, 25.5)	9 (2.7, 25.7)
Weight (kg)	8.8 (3.9, 15.8)	8.8 (3.8, 18.8)	9.5 (4.9, 15.8)	9.7 (4.7, 18.8)	8.2 (4.5, 13.5)	8.5 (5.2, 15.1)	9.1 (4, 14.8)	9 (3.8, 16)	7.6 (3.9, 12.7)	8 (5, 13.1)
Length (cm)	71.6 (52.3, 98.4)	72 (51.6, 92.8)	72.7 (54.8, 95.5)	72.5 (55.2, 92.1)	71 (53.9, 89.9)	72.6 (57.3, 92.8)	72.5 (52.3, 98.4)	73.4 (51.6, 90.6)	68.5 (55.2, 87.2)	69.5 (56.4, 91.6)
TSFT (mm)	9.2 (4.2, 16.6)	8.8 (4.5, 16.4)	9.6 (5.1, 14.3)	9.5 (5.8, 15.6)	8.5 (4.2, 12.1)	8.3 (4.6, 11.7)	10.1 (5.7, 16.6)	9.5 (5.1, 16.4)	7.4 (4.3, 15.2)	7.3 (4.5, 11.6)
SSFT (mm)	7.6 (4, 17.4)	7.3 (4.2, 17.1)	7 (4, 12.3)	7 (4.6, 13.9)	7.2 (5.1, 11.4)	6.9 (5.1, 9.8)	9 (5.3, 17.4)	8.7 (4.2, 17.1)	7 (4, 12.5)	6.6 (4.3, 14.2)
Head circumference (cm)	45.2 (35.2, 52.4)	45.3 (37.4, 51.4)	45.9 (37.4, 52.1)	46.4 (38.4, 51.4)	44.1 (37.5, 49.9)	44.7 (38.1, 50.3)	45.9 (35.2, 52.4)	45.8 (37.4, 50.8)	43.4 (37.4, 49.6)	43.9 (38.2, 49)
Arm circumference (cm)	14.6 (10.5, 18.6)	14.5 (10.3, 20.6)	15 (11.4, 18.6)	15.2 (11.1, 20.6)	14 (11.2, 16.8)	14.1 (11.9, 16.5)	14.6 (10.5, 18.2)	14.3 (10.3, 18.4)	14.5 (10.9, 17.8)	14.8 (12.6, 17.9)
FM (kg)	1.9 (0.2, 7.7)	1.8 (0.4, 6.9)	2 (0.2, 4.6)	1.9 (0.4, 6.9)	1.6 (0.6, 7.7)	1.6 (0.6, 3.6)	2.5 (0.7, 5.2)	2.4 (0.6, 5.6)	1.2 (0.3, 4.2)	1.3 (0.4, 2.8)
FFM (kg)	6.7 (3, 12.6)	6.9 (3.3, 12.3)	7.5 (3.9, 12.3)	7.5 (4, 12.3)	6.6 (3.8, 11.1)	6.7 (4, 12)	6.6 (3, 12.6)	6.6 (3.3, 10.7)	6.2 (3.6, 11.1)	6.6 (3.7, 10.9)
Girls										
Number of observations	942	441	323	150	182	105	296	124	141	62
Age (mo)	9.1 (2.5, 25.2)	9.1 (2.1, 25.9)	9.1 (2.7, 25.2)	10.4 (2.7, 25.4)	9.1 (2.9, 25.2)	9.1 (2.9, 25.9)	11.4 (2.5, 24.4)	9.3 (2.5, 24.2)	9 (2.7, 24.6)	6.3 (2.1, 24)
Weight (kg)	8.2 (4, 15.8)	8.1 (3.8, 15.6)	8.9 (4.7, 15.1)	8.8 (4.7, 15.6)	8.1 (4.6, 13.4)	7.7 (3.8, 14.8)	8.6 (4, 15.8)	8.5 (5.1, 13.6)	7.2 (4, 12.1)	6.8 (4.4, 11)
Length (cm)	70.1 (52.6, 93.3)	69.8 (52.4, 95.7)	70.8 (54.5, 93.3)	71.1 (55.4, 95.7)	70.1 (54.9, 89.7)	70.6 (53.2, 92)	70.7 (52.6, 91)	70.4 (52.4, 90.3)	66.2 (53, 89.2)	65.2 (55.5, 85.2)
TSFT (mm)	9.1 (4.4, 17.3)	9.3 (5, 18.7)	9.6 (5.6, 14)	9.7 (5.4, 16.5)	8.7 (5, 13.1)	8.3 (5, 13.3)	9.6 (4.8, 17.3)	9.7 (5.1, 18.7)	7.3 (4.4, 13.6)	7.7 (5.1, 12.6)
SSFT (mm)	7.7 (4.2, 15.8)	7.6 (4.5, 16.3)	7.2 (4.2, 12.3)	7 (4.6, 11.4)	7.7 (5, 14.2)	7.3 (5.1, 12.2)	8.9 (4.8, 15.8)	8.8 (4.5, 16.3)	7.3 (4.4, 13.5)	7.1 (4.9, 13.1)
Head circumference (cm)	43.9 (34.5, 49.8)	43.8 (36.8, 50.9)	44.5 (37.4, 49.4)	44.8 (37.9, 50.9)	43 (37.1, 49.5)	43.4 (37.1, 48.5)	44.5 (34.5, 49.8)	44.3 (38.3, 49.6)	42.7 (36.6, 48.8)	41.6 (36.8, 46.9)
Arm circumference (cm)	14.3 (10.5, 18.2)	14.4 (11, 18.4)	14.9 (11.1, 18.1)	14.6 (11.2, 18.4)	13.9 (11.2, 16.9)	13.8 (11, 17)	14.2 (10.5, 18.2)	14.5 (11.1, 18.2)	14.5 (11.6, 17.1)	14.4 (11.6, 16.9)
FM (kg)	1.9 (0.2, 5.6)	1.9 (0.3, 4.6)	1.9 (0.2, 4.1)	2 (0.3, 4.2)	1.8 (0.5, 4.4)	1.7 (0.5, 4.5)	2.4 (0.6, 5.6)	2.6 (1, 4.6)	1.3 (0.3, 4.4)	1.4 (0.3, 3.3)
FFM (kg)	6.2 (3.1, 12.1)	6.1 (3.3, 12.1)	6.8 (3.9, 12.1)	6.7 (3.7, 12.1)	6 (3.3, 10.6)	6.3 (3.3, 11.2)	6 (3.1, 11)	6 (3.6, 10.1)	5.8 (3.3, 11.2)	5.5 (3.4, 9.4)

Results are presented as median (range).

*FM* Fat mass, *FFM* Fat-free mass, *SSFT* Subscapular skinfold thickness, *TSFT* Triceps skinfold thickness.

**Table 2:** Comparison of age, anthropometric and body composition measurements in the test samples between 3 and 6 mo of age for each sex, pooled and for each country.

	Pooled	Australia	India	South Africa
	Test	Test	Test	Test
Boys				
Number of observations	164	30	46	88
Age (mo)	5.7 (2.8, 6.8)	5.8 (5.4, 6.8)	6.1 (5.3, 6.6)	3.6 (2.8, 6.2)
Weight (kg)	6.9 (4.8, 9.8)	7.4 (6.4, 9.1)	7.5 (6.2, 9.8)	6.6 (4.8, 9.2)
Length (cm)	64.6 (55.7, 75.3)	66.4 (62.5, 70.8)	66.9 (62, 71)	61.1 (55.7, 75.3)
TSFT (mm)	8.5 (4.2, 16.2)	12.7 (7.3, 16.2)	6.2 (4.2, 9.2)	9.2 (5.5, 14.7)
SSFT (mm)	8 (4.1, 16)	7.5 (4.7, 12.3)	6.8 (4.8, 9)	8.5 (4.1, 16)
FM (kg)	1.8 (0.8, 3.7)	1.8 (0.9, 3.2)	1.9 (0.8, 3.4)	1.8 (0.9, 3.7)
FFM (kg)	5.1 (3.5, 7.3)	5.7 (4.3, 6.7)	5.6 (4.6, 7.3)	4.7 (3.5, 6)
Girls				
Number of observations	185	21	44	120
Age (months)	5.5 (2.6, 6.6)	5.8 (5.5, 6.5)	6.1 (5.8, 6.6)	3.9 (2.6, 6.2)
Weight (kg)	6.6 (4.4, 9.9)	6.9 (6.1, 8)	6.9 (5.5, 8.7)	6.3 (4.4, 9.9)
Length (cm)	62.2 (52.4, 70.3)	64.2 (60.9, 68.1)	65.1 (61.8, 70.3)	60.3 (52.4, 69.9)
TSFT (mm)	8.5 (4, 18.4)	12.1 (8.4, 18.4)	5.9 (4, 9.8)	8.8 (5.3, 14.3)
SSFT (mm)	8.1 (4.8, 15.1)	7.1 (5.7, 10.4)	6.4 (4.8, 9.4)	8.8 (5.7, 15.1)
FM (kg)	1.9 (0.7, 4.5)	1.7 (0.9, 2.6)	1.9 (0.9, 3.3)	1.9 (0.7, 4.5)
FFM (kg)	4.7 (3.1, 6.2)	5.2 (4.5, 5.9)	4.8 (3.1, 6.2)	4.5 (3.3, 5.6)

Results are presented as median (range).

*FM* Fat mass, *FFM* Fat-free mass, *SSFT* Subscapular skinfold thickness, *TSFT* Triceps skinfold thickness.

### Developing the prediction equations

A priori power analysis was not conducted for developing the prediction equations and therefore included longitudinal data of all infants (*n* = 650, observations = 1896) for whom the data was available. Previous studies that estimated body composition using anthropometry had similar or smaller sample sizes [11]. Joint distribution of all variables was examined using scatterplots and Pearson correlations.

A linear mixed model on the training data separately for girls and boys, using linear splines with knots at 9 and 18 months, was used to develop the prediction equations for FM (kg) and FFM (kg). We used linear mixed models with random intercepts to account for clustering of observations among individuals. The knots for age were selected based on visual inspection of trajectories for FM and FFM. Additionally, the models were adjusted for length (m), WFL (kg/m), TSFT, SSFT, and Asian ethnicity. Adjusting for head and arm circumference did not influence the results, and as such, were not included in the final prediction equations. South Asian ethnicity was ascribed based on the country from which the participant was recruited. Estimation of the 95% prediction interval was attained by incorporating uncertainty in random effects (for training data only), uncertainty in fixed effects, and residual variance of outcome variable. A detailed description of the methodology is provided in Supplementary Note 1.

Subsequently, the fitted model was internally validated on the ‘validation’ data and evaluated for the quality of predictions using: (a) error metrics (root mean squared error - RMSE; root mean square percentage error – RMSPE; mean absolute error – MAE; and mean absolute percentage error - MAPE) for the predicted values, and (b) the number of instances for which true values were outside the prediction interval. A summary of the different error metrics used is provided in Supplementary Note 2.

### Validation of prediction equations

The fitted model was externally validated on the test data and evaluated for quality of predictions using the same error metrics as above. Additionally, systematic error in predictions were explored (on test data with observed and predicted values) using Bland-Altman plots.

### Sensitivity analysis

First, model misspecification was assessed by repeating the analysis with models fitted (a) using quadratic term for age, (b) using natural cubic splines for age, and (c) using natural cubic splines for all predictors. Natural splines are a family of piecewise cubic polynomials and were fitted with four degrees of freedom. These models were then compared using error metrics and the conditional Akaike Information Criterion [26]. Second, prediction error was assessed against the logarithmic transformation of the outcome variables for the four model specifications due to observed right skew in the outcome variables. Third, prediction error was assessed after substituting WFL as a predictor with (a) BMI (kg/m^2^) and (b) ponderal index (kg/m^3^) for the linear spline model. All analyses were executed using R 3.6.1 using lme4 (v1.1-23), merTools (v0.5.2) and cAIC4 (v1.0) packages.

## Results

**Fig. 1:**
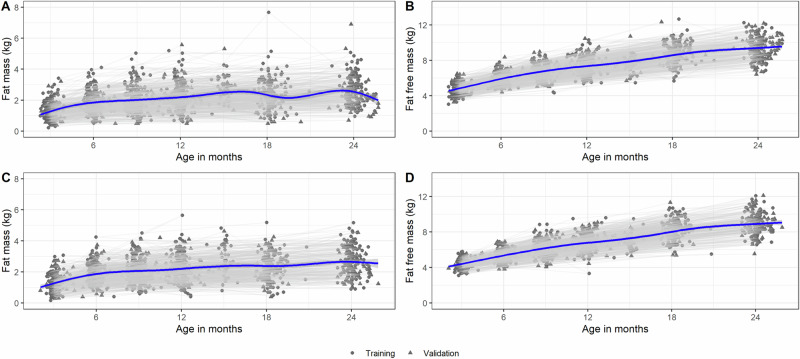
The trajectories of fat mass and fat free mass in children aged 3–24 months from low and middle income countries according to sex. All are in kg; **A** male fat mass, **B** male fat-free mass, **C** female fat mass, **D** female fat-free mass. The thick lines are loess smoothed trajectories fit using penalized cubic splines for all participants (*N*) in the Training and Validation data.

The distribution of FM and FFM by age at each visit stratified by cohort for each participant in the training data set of the four countries in the main cohorts are depicted in Fig. 1. The variance of FFM increased with age. We also provide the distribution of FM and FFM by cohort for training and validation data (Supplementary Fig. 2) and for test data (Supplementary Fig. 3).

### Association of anthropometry with FM and FFM

Unadjusted non-linear associations of age with FM and FFM were observed in both boys (Supplementary Fig. 4) and girls (Supplementary Fig. 5). Further, linear associations of length and weight-for-age with FM and FFM, were also observed. All anthropometric variables were positively correlated with FM and FFM (except skinfold thickness measures with FFM). The associations of skinfold thickness with FM and FFM were non-linear at higher values, potentially due to high leverage points.

After adjusting for other covariates, both length and WFL were positively associated with FM and FFM in boys and girls (Table 3). Both measures of skinfolds were independently and positively associated with FM, while they were negatively associated with FFM in boys and girls. The association of age with FM was non-linear from 9 to 18 month with a rapid reduction in slope (Boys: −0.04 kg per month, 95%CI: −0.07, −0.01; Girls: −0.05 kg per month, 95%CI: −0.08, −0.02). The association was also non-linear after 18 months, with an offset in the slope reduction from an increase in slope (boys: 0.03 kg per month, 95%CI: 0.00, 0.06; girls: 0.06, 95%CI: 0.03, 0.09). Similarly, the association of age with FFM after adjusting for other covariates was non-linear, with a negative slope up to 9 months, a rapid increase in slope from 9 to 18 months and a subsequent rapid reduction in the slope.

**Table 3:** Coefficients for linear spline model.

in kg (95% CI)	Fat mass		Fat-free mass	
	Male (*n* = 954)	Female (*n* = 942)	Male (*n* = 954)	Female (*n* = 942)
Intercept	−4.05 (−4.84, −3.26)	−3.66 (−4.44, −2.88)	−4.68 (−5.49, −3.87)	−4.46 (−5.25, −3.66)
Non-Asian	0.19 (0.09, 0.29)	0.2 (0.1, 0.31)	−0.23 (−0.34, −0.13)	−0.21 (−0.32, −0.1)
Age (mo)	0.01 (−0.02, 0.04)	0.02 (−0.01, 0.05)	−0.06 (−0.09, −0.03)	−0.06 (−0.1, −0.03)
Age-9 (mo)	−0.04 (−0.07, −0.01)	−0.05 (−0.08, −0.02)	0.1 (0.07, 0.13)	0.11 (0.08, 0.14)
Age-18 (mo)	0.03 (0, 0.06)	0.06 (0.03, 0.09)	−0.03 (−0.06, 0.01)	−0.05 (−0.08, −0.02)
Length (m)	2.42 (0.96, 3.89)	2.68 (1.22, 4.15)	10.21 (8.71, 11.71)	9.72 (8.23, 11.21)
WFL(kg/m)	0.25 (0.21, 0.29)	0.2 (0.16, 0.24)	0.47 (0.43, 0.51)	0.48 (0.43, 0.52)
TSFT (mm)	0.05 (0.02, 0.07)	0.04 (0.01, 0.06)	−0.03 (−0.05, 0)	−0.02 (−0.04, 0)
SSFT(mm)	0.09 (0.06, 0.11)	0.1 (0.08, 0.13)	−0.1 (−0.13, −0.08)	−0.12 (−0.15, −0.09)
*Marginal R* ^*2*^	0.6	0.56	0.9	0.89
*Conditional R* ^*2*^	0.66	0.67	0.91	0.91

All values displayed are regression coefficients (in kg) and 95% confidence intervals.

The number of observations is for the complete-case dataset without missing values on the above explanatory variables and outcomes.

*SSFT* Subscapular skinfold thickness, *TSFT* Triceps skinfold thickness, *WFL* Weight-for- length.

The equations to predict FM and FFM for each sex are provided in Table 4. RMSE was higher for FM and FFM among older children in both boys (FM: 0.66; FFM: 0.66) and girls (FM: 0.51; FFM: 0.53). RMSE was also similar between training and validation for older age. In the assessment of FM and FFM of the 3−9-month age group, the RMSE was also similar between validation and test data (Table 4). Most observations were within the prediction intervals which incorporated uncertainty in fixed effects and residual variance, for validation and test data (Table 5). RMSPE and MAPE were higher for validation data while predicting FM in males and females, compared to test data. RMSPE and MAPE were also higher in test data for predicting FFM in males and females, compared to validation data. RMSE was under 510 g for prediction of FM in validation group, but RMSPE was very high. MAE in the prediction of FFM was in the range of 350 to 460 g in all three groups, but the MAPE was low (under 10%).

**Table 4:** Prediction equations for fat and fat-free mass in children 3 to 24 months.

		Training		Validation		Test	
		RMSE (kg)	*r*	RMSE (kg)	*r*	RMSE (kg)	*r*
Ages (mo)	Male FM	0.48	0.84	0.51	0.81	0.55	0.57
3–9	FM = –4.05 + 0.19*Non-South Asian + 0.01*Age + 2.42*Length + 0.25*WFL + 0.05*TSFT + 0.09*SSFT	0.36	0.87	0.47	0.76	0.55	0.57
10–18	FM = –4.05 + 0.19*Non-South Asian + 0.01*Age – 0.04*(Age – 9) + 2.75*Length + 0.25*WFL + 0.05*TSFT + 0.09*SSFT	0.49	0.82	0.54	0.79	-	-
19–24	FM = –4.05 + 0.19*Non-South Asian + 0.01*Age – 0.04*(Age – 9) + 0.03*(Age – 18) + 2.42*Length + 0.25*WFL + 0.05*TSFT + 0.09*SSFT	0.66	0.75	0.54	0.83	-	-
	Female FM	0.46	0.85	0.49	0.82	0.52	0.67
3–9	FM = –3.66 + 0.20*Non-South Asian + 0.02*Age + 2.68*Length + 0.20*WFL + 0.04*TSFT + 0.10*SSFT	0.38	0.84	0.47	0.8	0.52	0.67
10–18	FM = –3.66 + 0.20*Non-South Asian + 0.02*Age – 0.05*(Age – 9) + 2.68*Length + 0.20*WFL + 0.04*TSFT + 0.10*SSFT	0.52	0.81	0.48	0.82	-	-
19–24	FM = –3.66 + 0.20*Non-South Asian + 0.02*Age – 0.05*(Age – 9) + 0.06*(Age –18) + 2.68*Length + 0.20*WFL + 0.04*TSFT + 0.10*SSFT	0.51	0.85	0.55	0.76	-	-
	Male FFM	0.48	0.96	0.51	0.96	0.57	0.78
3–9	FFM = –4.68 – 0.23*Non-South Asian – 0.06*Age + 10.21*Length + 0.47*WFL –0.03*TSFT – 0.10*SSFT	0.38	0.92	0.47	0.89	0.57	0.78
10–18	FFM = –4.68 – 0.23*Non-South Asian – 0.06*Age + 0.10*(Age – 9) + 10.21*Length + 0.47*WFL –0.03*TSFT – 0.10*SSFT	0.48	0.9	0.55	0.88	-	-
19–24	FFM = –4.68 – 0.23*Non-South Asian – 0.06*Age + 0.10*(Age – 9) – 0.03*(Age–18) + 10.21*Length + 0.47*WFL –0.03*TSFT – 0.10*SSFT	0.66	0.87	0.54	0.91	-	-
	Female FFM	0.47	0.96	0.5	0.95	0.54	0.77
3–9	FFM = –4.46 – 0.21*Non-South Asian – 0.06*Age + 9.72*Length + 0.48*WFL –0.02*TSFT – 0.12*SSFT	0.4	0.91	0.49	0.86	0.54	0.77
10–18	FFM = –4.46 – 0.21*Non-South Asian – 0.06*Age + 0.11*(Age – 9) + 9.72*Length + 0.48*WFL –0.02*TSFT – 0.12*SSFT	0.52	0.89	0.47	0.91	-	-
19–24	FFM = –4.46 – 0.21*Non-South Asian – 0.06*Age + 0.11*(Age – 9) – 0.05*(Age–18) + 9.72*Length + 0.48*WFL –0.02*TSFT – 0.12*SSFT	0.53	0.9	0.55	0.92	-	-

‘Non-Asian’ is an indicator variable that takes value of 0 for Asian population and 1 for Non-Asian population.

*FM* Fat mass (kg), *FFM* Fat-free mass (kg), Age (mo), Length (m), *SSFT* subscapular skin fold thickness (mm), *TSFT* Triceps skin fold thickness.

(mm), *WFL* Weight-for-length (kg/m), *r* Pearsons correlation.

**Table 5:** Estimates of uncertainty in prediction of fat and fat-free mass in training, validation and test groups.

Dataset	Total observations	Observations outside	Pearson correlation	RMSE (kg)	MAE (kg)	RMSPE (%)	MAPE (%)
FM in males							
Training	954	32 (3.4%)	0.84	0.48	0.35	35.2	21.4
Validation	500	28 (5.6%)	0.81	0.51	0.4	42.9	27.1
Test	164	11 (6.7%)	0.57	0.55	0.45	29.1	24.3
FM in females							
Training	942	25 (2.7%)	0.85	0.46	0.35	35.6	21.8
Validation	441	22 (5.0%)	0.82	0.49	0.39	40.8	24.3
Test	185	11 (5.9%)	0.67	0.52	0.41	26.8	21.8
FFM in males							
Training	954	26 (2.7%)	0.96	0.48	0.35	7.1	5.2
Validation	500	19 (3.8%)	0.96	0.51	0.41	7.5	6
Test	164	11 (6.7%)	0.78	0.57	0.46	11.3	9.1
FFM in females							
Training	942	27 (2.9%)	0.96	0.47	0.36	7.9	5.8
Validation	441	20 (4.5%)	0.95	0.5	0.39	8.6	6.4
Test	185	9 (4.9%)	0.77	0.54	0.44	12.2	9.5

The 95% prediction interval for validation and test data incorporates uncertainty in fixed effects and residual variance of outcome variable using 1000 simulations (Supplementary Note 1). The formula for different error metrics is provided in Supplementary Note 2. Estimates for each country is provided in Supplementary Table 1.

*FM* Fat mass, *FFM* Fat free mass, *MAE* Mean absolute error, *MAPE* Mean absolute percentage error, *RMSE* Root mean squared error, *RMSPE* Root mean squared percentage error.

When the RMSPE for assessment of FM predictions were evaluated by country, Sri Lanka showed a higher value (boys 47.1, girls 55.2) (Supplementary Table 1) compared to the mean value for training group (boys 35.2, girls 35.6) and validation group (boys 42.9, girls 40.8) (Table 5). Similarly, MAPE values for Sri Lanka (boys 33.7 girls 36.3) were higher compared to the mean value for training group (boys 21.4, girls 21.8) and validation group (boys 27.1, girls 24.3). For FFM, Sri Lanka RMSPE values (boys 7.4, girls 9.1) were similar to mean training group values (boys 7.1, girls 7.9) and mean validation group values (boys 7.5, girls 8.6). Similarly, MAPE values for Sri Lankan children (boys 5.7, girls 6.4) were comparable with mean training group values (boys 5.2, girls 5.8) and mean validation group values (boys 6.0, girls 6.4).

Of the three countries in the test group for assessment of FFM, RMSPE for South Africa (boys 9.9, girls 10.1), Australia (boys 10.0, girls 6.2) and India (boys 14.3 girls 18.2) (Supplementary Table 1) showed good agreement with the validation group (boys 7.5, girls 8.6) (Table 5). Similarly, MAPE for South Africa (boys 7.8, girls 8.2), Australia (boys 7.9, girls 4.9) and India (boys 12.5, girls 15.4) also showed a similar distribution as the validation group (boys 6.0, girls 6.4).

**Fig. 2:**
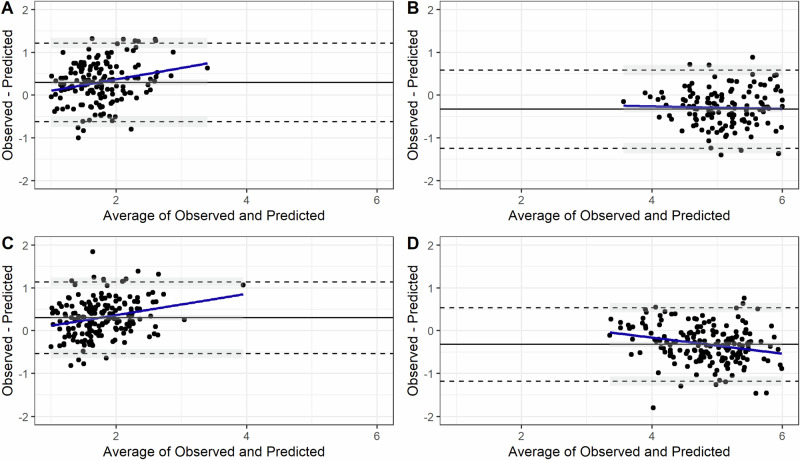
The Bland–Altman plots for prediction of fat mass and fat free mass on test data from Australia, India and South Africa children. All are in kg; the difference (**d**) of mean line (solid black) from zero is the systematic difference between measurements (i.e., estimated bias). Points outside the limits of agreement (±1.96 SD; dashed black) lines are outlier predictions. Correlation (*r*) in the Bland–Altman plot between average and difference suggests proportional bias such that the prediction model doesn’t agree over the range of outcome values. **A** Male fat mass (*n* = 164; *d* = 0.29, *r* = 0.24, *p* < 0.01), **B** male fat-free mass (*n* = 164; *d* = −0.33, *r* = −0.09, *p* = 0.21), **C** female fat mass (*n* = 185; *d* = 0.30, *r* = 0.27, *p* < 0.01), **D** female fat-free mass (*n* = 185; *d* = −0.32, *r* = −0.26, *p* < 0.01).

The Bland-Altman plot for predictions on the test data (Fig. 2) suggested no proportional bias for FFM in males. The model for FFM in males underestimated the outcome by 0.33 kg. The plot was suggestive of proportional bias (|r| ≈ 0.3) for FM in males and females, and for FFM in females.

### Sensitivity analysis

Comparison of alternate model specifications against the linear spline model showed similar error in predicting the outcomes in the training and validation data for both FM and FFM in males and females (data not shown). The conditional AIC for the linear spline model was better than the model with quadratic age (Supplementary Table 2). However, the models with natural splines fit for all predictors had the best fit for both outcomes in males and females. Log-transforming the outcome yielded similar errors in prediction as the model without transforming the outcome. Similarly, using an alternate version of weight adjusted for length (BMI, ponderal index) did not yield different results from using WFL in the linear spline model (data not shown**)**.

## Discussion

This research enabled the generation of four sets of anthropometry-based equations for the prediction of FM and FFM in male and female infants from diverse ethnic and socio-economic settings, in three different age categories.

To the best of our knowledge, this is the first multi-ethnic equation developed on a population representing different regions of the world, to evaluate body composition in the first 24 months of life. Validation of the equations showed good correlation between actual and predicted values. The study group was divided broadly into South Asian and non-South Asian ethnicity and is likely to be a logical division as most prior data have shown a significant difference between South Asian ethnicity and others.

Apart from validating the equations on a subsample of the same cohort, the 3–9-month age range equation was applied on a different cohort of children derived from three socioeconomically and ethnically diverse populations. RMSE and MAE for test data were quite similar to the training and validation data, and the numbers of observations that were seen outside the prediction interval were also similar. Equations had a better prediction value for FFM compared to FM. Thus, these equations might be better used to predict FFM, with calculation of FM by subtracting FFM from weight, rather than by directly assessing FM.

Regression models with diverse variables can be used as predictors of body composition during the first two years of life. The value of this approach has been shown previously by Gopalakrishnamoorthy and co-workers based on multi-ethnic population derived anthropometry-based equations for the assessment of body fat at 3 days and 15 weeks for both girls and boys, and a sex combined equation at 54 weeks of age using weight, length, head circumference and skinfold thicknesses, with a high coefficient determination (R^2^) (0.82–0.92) [27]. As sex and age during the first two years affect body composition, our study also provided sex-specific equations for the assessment of FM and FFM for three different age categories. Length, WFL, TSFT, SSFT and Asian ethnicity showed clear association with FM and FFM, although some studies have shown skinfold thicknesses not to have predictive value of total body fat [28]. We used both TSFT and SSFT as variables, which improved the predictability of FM, and the predictability was higher for males. Length and head circumference are determined more by genetics than by nutrition [29, 30]. Our prediction model did not improve by adjusting for head circumference and arm circumference.

A systematic review revealed that age, ethnicity and socioeconomic status affect the body composition of infants [31]. With physical growth, body composition changes from birth through infancy to childhood [32]. With age, the non-subcutaneous FM increases, with higher values seen in females [28]. These tissue changes underline the importance of using prediction equations suitable for different ages and sex [14]. Furthermore, ethnic differences in body composition have been noted [31, 33]. In the London mother and baby study, South Asian ethnicity had less FFM and more FM compared to European ancestry at 6–12 weeks of age and authors concluded that it is a reflection of ethnicity or maternal physiology during intrauterine life rather than dietary or behavioural factors [33, 34]. Developing reference data accounting for age and ethnic diversity provides a more meaningful understanding and assessment of body composition.

As stipulated in the Developmental Origins of Health and Diseases (DOHaD) hypothesis, the first 1000 days of life is closely linked with prospective health outcomes in later life. Details of body composition provide a much better understanding of physical growth related to health than anthropometry alone. Higher birth weight, a rapid gain in weight, especially accretion of fat during the first 1000 days of life, predisposes an individual to develop obesity in childhood, and assessment of body composition would help in getting a better assessment and taking steps in prevention [35]. Anthropometry-based equations have gained popularity over time due to their reasonable accuracy and ease of application and cost-effectiveness in low resource settings. Indirect body composition assessment techniques are not freely available for the first twenty-four months of life and the sex-specific prediction equations provided by this study for children from 3 to 24 months, developed on data from several countries of different socioeconomic backgrounds and ethnicities, would be a useful tool.

Many factors affect the results of prediction equations, and one of the most important is to apply a prediction equation on populations that are similar to the ones used in the original development. Even if age and sex are matched, they will still not produce accurate results if socioeconomic and ethnic background are not matched [14]. The test group was multi-ethnic and South Africa was part of both training and test groups. After desegregation of test data by individual country, test results showed good agreement with validation data for assessment of FFM. This affirms the notion that equations developed on the similar population are more applicable than those introduced from a different population.

A major strength of this study is that it used a multi-ethnic population of different socio-economic strata and equations have been cross validated on an independent group of infants with different socio-economic backgrounds. In conclusion, this study provides anthropometry-based equations for the prediction of FM and FFM for three age categories within the first 24 months of life for each sex. Predictability of FFM was higher than predictability of FM. Due to its wide representation, these equations would be better than previously developed equations and help in the assessment of body composition during early years of life easily and accurately.

## Supplementary information

Supplementary Figure Legends

Supplementary Figure 1

Supplementary Figure 2

Supplementary Figure 3

Supplementary Figure 4

Supplementary Figure 5

Supplementary table 1

Supplementary table 2

Supplementary Note 1

Supplementary Note 2

## Data Availability

Analysis scripts are available at https://github.com/jvargh7/child_body_composition.
