# Supplementary material for: Anthropometric prediction models of body composition in 3 to 24month old infants: a multicenter international study
Source: Eur J Clin Nutr. 2024 Sep 20;78(11):943–51. doi: 10.1038/s41430-024-01501-0 (PMC11537960; doi:10.1038/s41430-024-01501-0)
Supplement: Supplementary file 1 — Supplementary Figure Legends [file 41430_2024_1501_MOESM1_ESM.docx]

# Supplementary Figure Legends

# Supplementary Figure 1. Flowchart of participation of training, validation and test groups by country and sex

# Supplementary Fig 2. Distribution of fat mass and fat-free mass in training and validation data by age and sex of cohort of each country

# Supplementary Figure 3. Distribution of fat mass and fat-free mass in test data by age and sex of cohort of each country

Supplementary Figure 4. Joint distribution of outcomes and predictors in training data for males of all cohorts pooled together

Supplementary Fig 5. Joint distribution of outcomes and predictors in females of all cohorts pooled together
