# Supplementary material for: Anthropometric prediction models of body composition in 3 to 24month old infants: a multicenter international study
Source: Eur J Clin Nutr. 2024 Sep 20;78(11):943–51. doi: 10.1038/s41430-024-01501-0 (PMC11537960; doi:10.1038/s41430-024-01501-0)
Supplement: Supplementary file 2 — Supplementary Figure 1 [file 41430_2024_1501_MOESM2_ESM.docx]

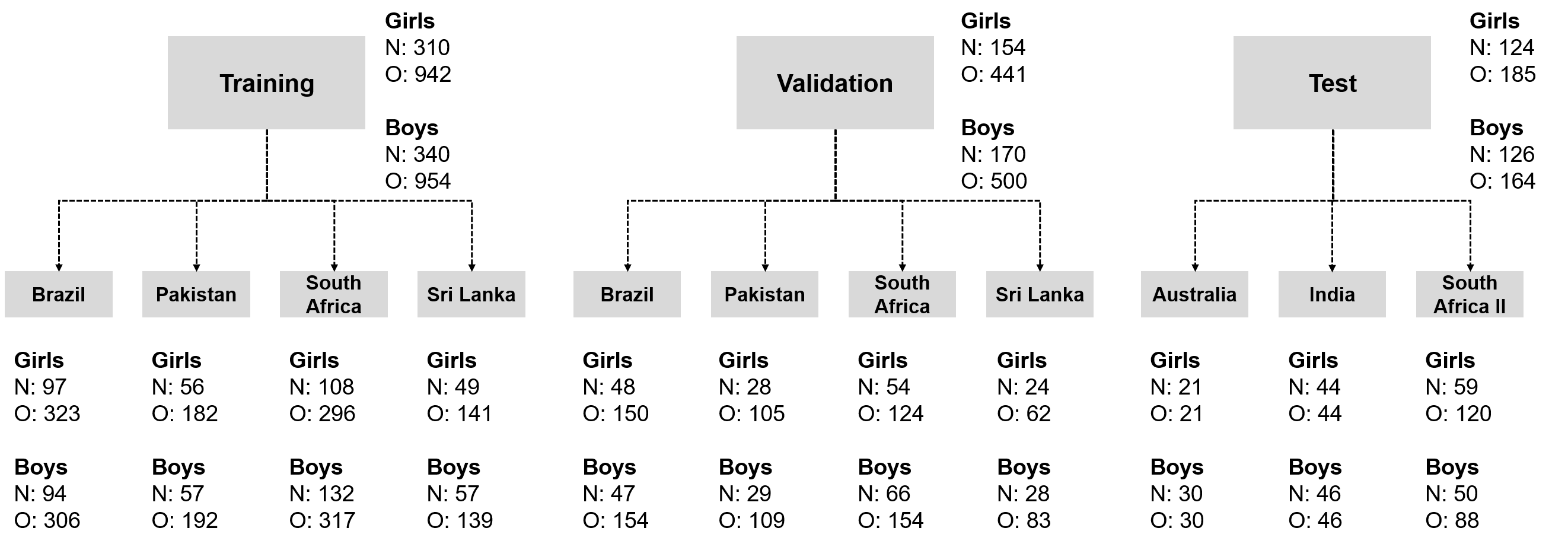


N: Number of participants, O: Number of total observations from all participants; We excluded one record each in males and females due to unavailability of subscapular skinfold thickness in the pooled dataset for training and validation. In the test dataset, excluded 5 observations in males and 6 observations in females due to unavailability of one of triceps skinfold and subscapular skinfold thickness.
