# Supplementary figures and images for: Anthropometric prediction models of body composition in 3 to 24month old infants: a multicenter international study
Source: Eur J Clin Nutr. 2024 Sep 20;78(11):943–51. doi: 10.1038/s41430-024-01501-0 (PMC11537960; doi:10.1038/s41430-024-01501-0)

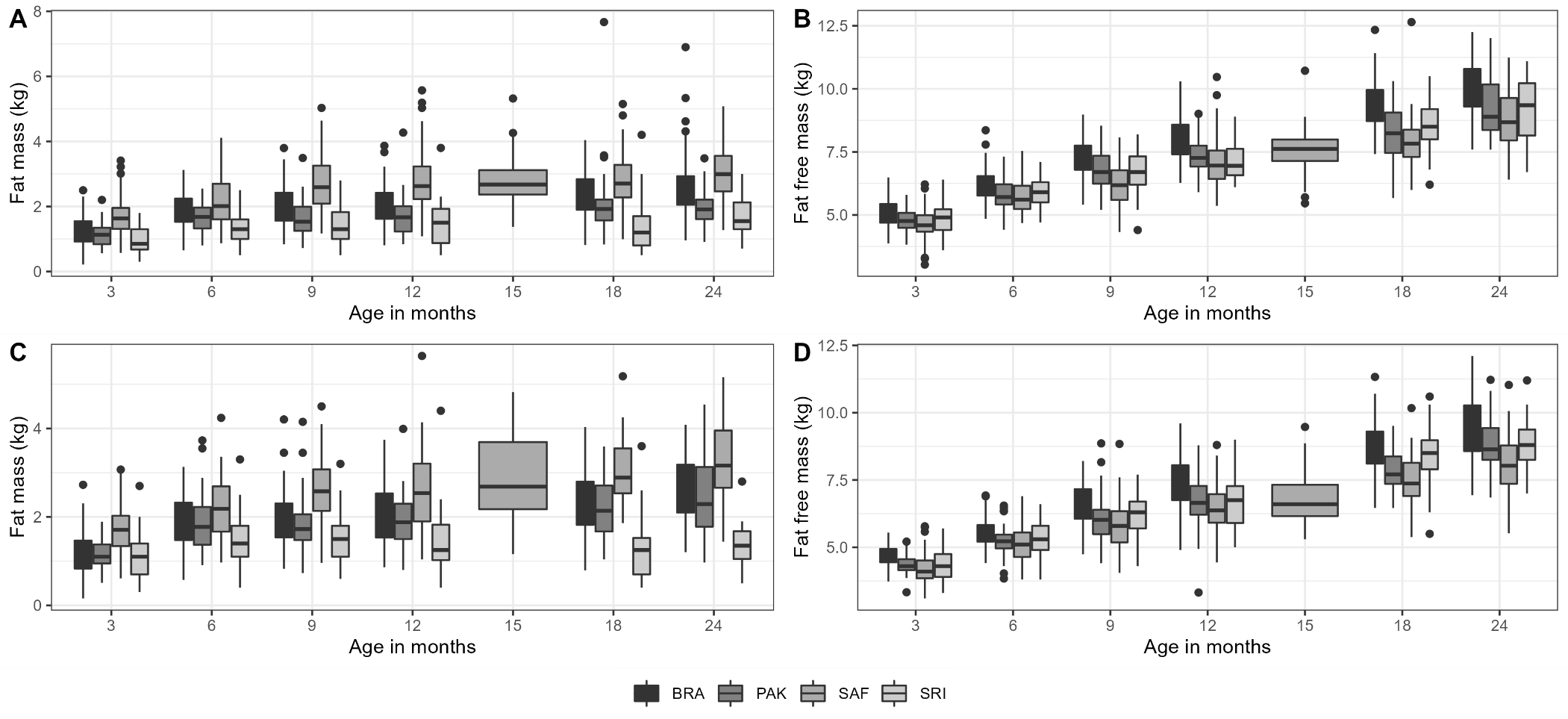


A: Male fat mass, B: Male fat free mass, C: Female fat mass, D: Female fat free mass

Supplement: Supplementary file 3 — Supplementary Figure 2 [file 41430_2024_1501_MOESM3_ESM.docx]

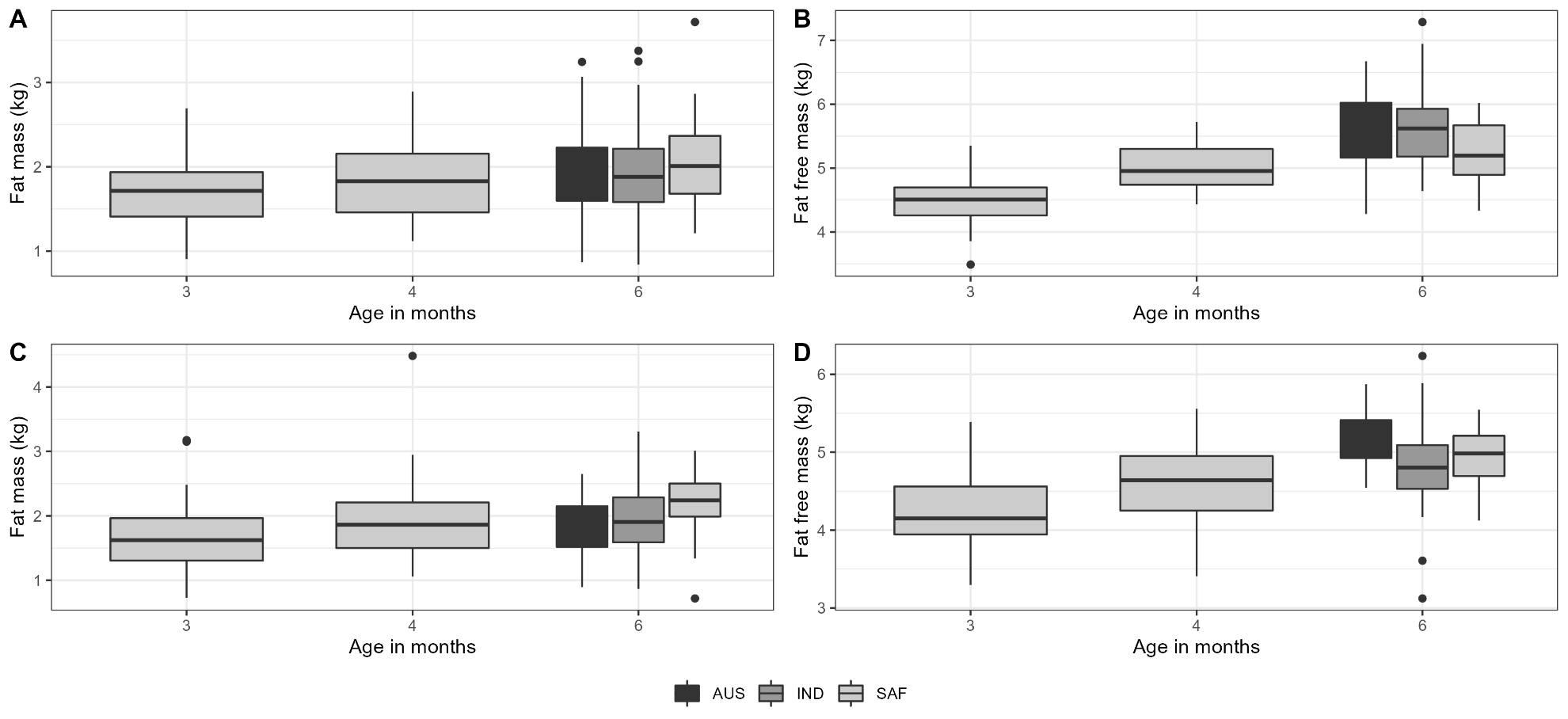


A: Male fat mass, B: Male fat free mass, C: Female fat mass, D: Female fat free mass

Supplement: Supplementary file 4 — Supplementary Figure 3 [file 41430_2024_1501_MOESM4_ESM.docx]
