# Supplementary material for: Anthropometric prediction models of body composition in 3 to 24month old infants: a multicenter international study
Source: Eur J Clin Nutr. 2024 Sep 20;78(11):943–51. doi: 10.1038/s41430-024-01501-0 (PMC11537960; doi:10.1038/s41430-024-01501-0)
Supplement: Supplementary file 5 — Supplementary Figure 4 [file 41430_2024_1501_MOESM5_ESM.docx]

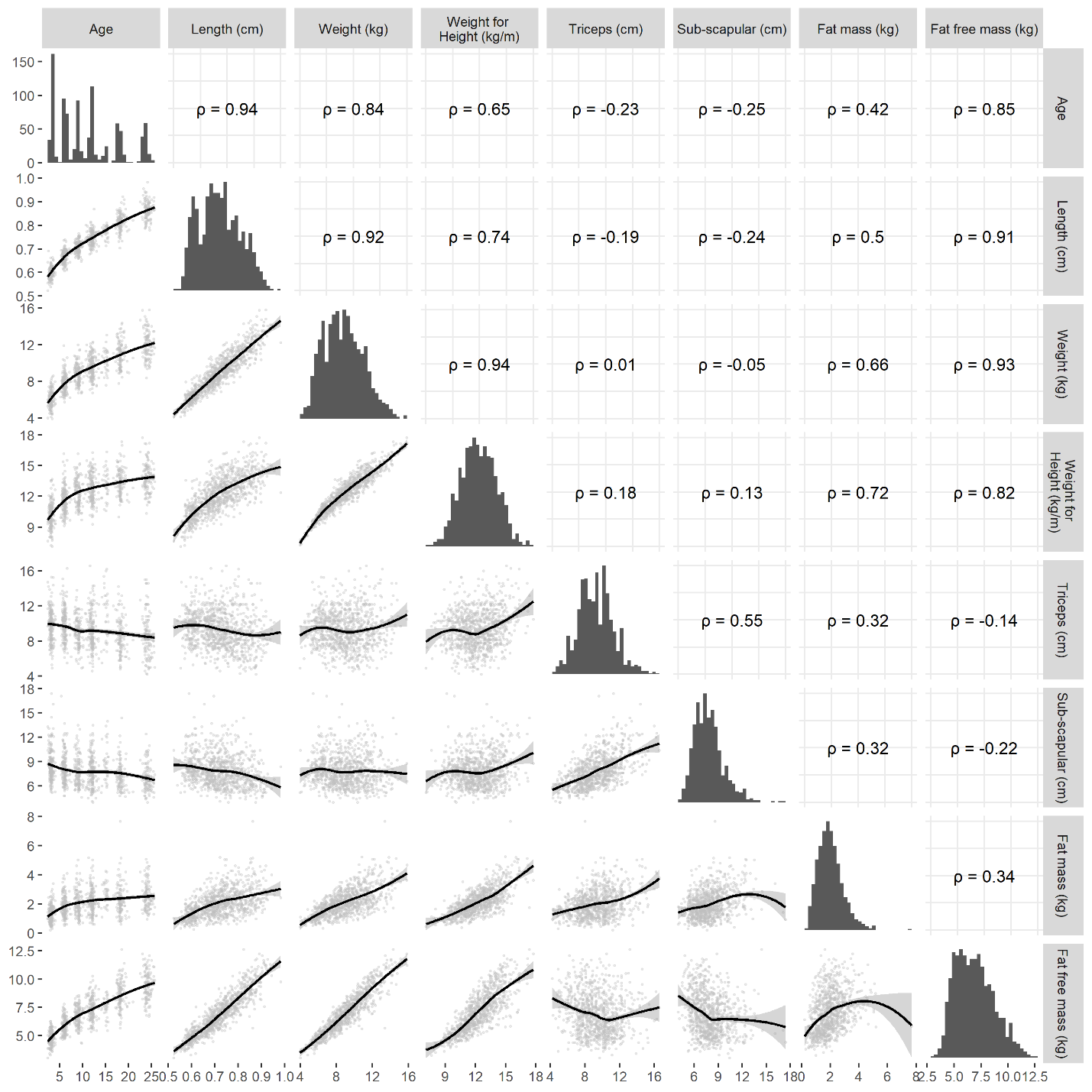


All values are in the pooled training set for males consisting of all observations from Brazil, Pakistan, South Africa and Sri Lanka. The black regression line in the scatterplot is the loess fit.
