# Supplementary material for: Anthropometric prediction models of body composition in 3 to 24month old infants: a multicenter international study
Source: Eur J Clin Nutr. 2024 Sep 20;78(11):943–51. doi: 10.1038/s41430-024-01501-0 (PMC11537960; doi:10.1038/s41430-024-01501-0)
Supplement: Supplementary file 7 — Supplementary table 1 [file 41430_2024_1501_MOESM7_ESM.docx]

**Supplementary Table 1: Estimates of uncertainty in prediction of FM and FFM split by countries of training, validation, and test groups**

| **Dataset** |  | **Total observations** | **Observations outside**  **prediction interval** | **Correlation** | **RMSE (kg)** | **MAE (kg)** | **RMSPE (%)** | **MAPE (%)** |
| --- | --- | --- | --- | --- | --- | --- | --- | --- |
| **FM in males** |  |  |  |  |  |  |  |  |
| Training | Brazil | 306 | 10 (3.3%) | 0.81 | 0.43 | 0.32 | 42.1 | 21.2 |
| Training | Pakistan | 192 | 2 (1%) | 0.76 | 0.47 | 0.3 | 24.8 | 19.2 |
| Training | South Africa | 317 | 16 (5%) | 0.83 | 0.53 | 0.4 | 26.2 | 17.6 |
| Training | Sri Lanka | 139 | 4 (2.9%) | 0.68 | 0.48 | 0.37 | 47.1 | 33.7 |
| Validation | Brazil | 154 | 14 (9.1%) | 0.83 | 0.57 | 0.43 | 49.6 | 30.3 |
| Validation | Pakistan | 109 | 2 (1.8%) | 0.71 | 0.4 | 0.31 | 27.8 | 20.6 |
| Validation | South Africa | 154 | 6 (3.9%) | 0.81 | 0.52 | 0.42 | 24.7 | 18.8 |
| Validation | Sri Lanka | 83 | 6 (7.2%) | 0.64 | 0.52 | 0.42 | 66.1 | 45.2 |
| Test | Australia | 30 | 1 (3.3%) | 0.47 | 0.5 | 0.42 | 32.3 | 24.8 |
| Test | India | 46 | 8 (17.4%) | 0.64 | 0.75 | 0.66 | 35.3 | 32.7 |

| Test | South Africa | 88 | 2 (2.3%) | 0.7 | 0.44 | 0.36 | 23.9 | 19.7 |
| --- | --- | --- | --- | --- | --- | --- | --- | --- |
| **FM in females** |  |  |  |  |  |  |  |  |
| Training | Brazil | 323 | 3 (0.9%) | 0.86 | 0.4 | 0.31 | 38.1 | 22 |
| Training | Pakistan | 182 | 6 (3.3%) | 0.81 | 0.44 | 0.32 | 24.4 | 17.8 |
| Training | South Africa | 296 | 8 (2.7%) | 0.85 | 0.5 | 0.39 | 25.2 | 17 |
| Training | Sri Lanka | 141 | 8 (5.7%) | 0.6 | 0.52 | 0.38 | 55.2 | 36.3 |
| Validation | Brazil | 150 | 7 (4.7%) | 0.81 | 0.47 | 0.38 | 47.1 | 26.6 |
| Validation | Pakistan | 105 | 3 (2.9%) | 0.83 | 0.43 | 0.34 | 27.6 | 21.2 |
| Validation | South Africa | 124 | 8 (6.5%) | 0.81 | 0.54 | 0.43 | 22.1 | 16.9 |
| Validation | Sri Lanka | 62 | 4 (6.5%) | 0.57 | 0.52 | 0.4 | 64.6 | 38.8 |
| Test | Australia | 21 | 0 (0%) | 0.7 | 0.34 | 0.24 | 30.3 | 17.6 |
| Test | India | 44 | 9 (20.5%) | 0.66 | 0.8 | 0.71 | 37.4 | 34.8 |
| Test | South Africa | 120 | 2 (1.7%) | 0.81 | 0.41 | 0.33 | 20.8 | 17.8 |
| **FFM in males** |  |  |  |  |  |  |  |  |
| Training | Brazil | 306 | 9 (2.9%) | 0.98 | 0.44 | 0.33 | 5.8 | 4.5 |
| Training | Pakistan | 192 | 1 (0.5%) | 0.96 | 0.47 | 0.31 | 7.6 | 4.7 |

| Training | South Africa | 317 | 12 (3.8%) | 0.94 | 0.53 | 0.4 | 7.8 | 6.2 |
| --- | --- | --- | --- | --- | --- | --- | --- | --- |
| Training | Sri Lanka | 139 | 4 (2.9%) | 0.96 | 0.48 | 0.36 | 7.4 | 5.7 |
| Validation | Brazil | 154 | 8 (5.2%) | 0.97 | 0.56 | 0.43 | 7.2 | 5.8 |
| Validation | Pakistan | 109 | 2 (1.8%) | 0.97 | 0.43 | 0.35 | 6.2 | 5 |
| Validation | South Africa | 154 | 7 (4.5%) | 0.95 | 0.53 | 0.43 | 8.6 | 6.9 |
| Validation | Sri Lanka | 83 | 2 (2.4%) | 0.95 | 0.5 | 0.41 | 7.4 | 6 |
| Test | Australia | 30 | 1 (3.3%) | 0.62 | 0.51 | 0.42 | 10 | 7.9 |
| Test | India | 46 | 8 (17.4%) | 0.64 | 0.77 | 0.68 | 14.3 | 12.5 |
| Test | South Africa | 88 | 2 (2.3%) | 0.73 | 0.46 | 0.36 | 9.9 | 7.8 |
| **FFM in females** |  |  |  |  |  |  |  |  |
| Training | Brazil | 323 | 5 (1.5%) | 0.97 | 0.43 | 0.34 | 6.2 | 4.9 |
| Training | Pakistan | 182 | 6 (3.3%) | 0.96 | 0.46 | 0.33 | 8.6 | 5.6 |
| Training | South Africa | 296 | 9 (3%) | 0.94 | 0.49 | 0.38 | 8.4 | 6.4 |
| Training | Sri Lanka | 141 | 7 (5%) | 0.96 | 0.53 | 0.38 | 9.1 | 6.4 |
| Validation | Brazil | 150 | 7 (4.7%) | 0.96 | 0.5 | 0.39 | 7.6 | 5.9 |
| Validation | Pakistan | 105 | 2 (1.9%) | 0.97 | 0.43 | 0.33 | 7.1 | 5.4 |

| Validation | South Africa | 124 | 8 (6.5%) | 0.95 | 0.53 | 0.42 | 9.6 | 7.2 |
| --- | --- | --- | --- | --- | --- | --- | --- | --- |
| Validation | Sri Lanka | 62 | 3 (4.8%) | 0.91 | 0.53 | 0.41 | 11 | 7.8 |
| Test | Australia | 21 | 0 (0%) | 0.58 | 0.33 | 0.26 | 6.2 | 4.9 |
| Test | India | 44 | 6 (13.6%) | 0.75 | 0.8 | 0.71 | 18.2 | 15.4 |
| Test | South Africa | 120 | 3 (2.5%) | 0.82 | 0.45 | 0.37 | 10.1 | 8.2 |

FM: Fat mass, FFM: Fat-free mass, MAE: Mean absolute error, MAPE: Mean absolute percentage error, RMSE: Root mean squared error, RMSPE: Root mean squared percentage error
