# Supplementary material for: Anthropometric prediction models of body composition in 3 to 24month old infants: a multicenter international study
Source: Eur J Clin Nutr. 2024 Sep 20;78(11):943–51. doi: 10.1038/s41430-024-01501-0 (PMC11537960; doi:10.1038/s41430-024-01501-0)
Supplement: Supplementary file 8 — Supplementary table 2 [file 41430_2024_1501_MOESM8_ESM.docx]

Supplementary Table 2. Comparison of conditional Akaike Information Criterion on training data for different model formulations

|  | Linear spline for  age | Quadratic age | Natural spline for  age | Natural spline for  all predictors |
| --- | --- | --- | --- | --- |
| FM in boys | 1559.3 | 1561.6 | 1559.4 | 1539.9 |
| FFM in boys | 1585.9 | 1599 | 1571 | 1570.7 |
| FM in girls | 1525.2 | 1541.5 | 1519.8 | 1525.8 |
| FFM in girls | 1557 | 1585.6 | 1538.9 | 1543.3 |

Model comparison for mixed models cannot be carried out using measures such as Bayesian Information Criterion or Akaike Information Criterion. Calculating BIC requires effective sample size and estimated number of parameters, that are not clearly defined in a mixed model context (1). AIC is not asymptotically unbiased and favors smaller models without random effects(2). A version of Conditional AIC (cAIC), that incorporates the estimation uncertainty in random effects covariance matrix, facilitates model selection(3).

The lower the value of cAIC, the better the model fit.

References:

1. Delattre M, Laviette, M., Poursat, M. A note on BIC in mixed-effects model. Electron J Statis. 2014;8(1):19
2. Greven S, Kneib, T. On the behaviour of marginal and conditional AIC in linear

mixed models. Biometrika. 2010;97(4):17.

1. Safken B, Rugamer, D., Kneib, T., Greven, S. Conditional Model Selection in

Mixed-Effects Models with cAIC4. Journal of Statistical Software. 2021;99(8):30.
