# Supplementary material for: Anthropometric prediction models of body composition in 3 to 24month old infants: a multicenter international study
Source: Eur J Clin Nutr. 2024 Sep 20;78(11):943–51. doi: 10.1038/s41430-024-01501-0 (PMC11537960; doi:10.1038/s41430-024-01501-0)
Supplement: Supplementary file 9 — Supplementary Note 1 [file 41430_2024_1501_MOESM9_ESM.docx]

# Supplementary Note 1. Expanded methodology of linear and cubic splines

***Splines***

Most associations between an exposure and outcome in the real-world are non-linear. The easiest of non-linear regression models for a continuous outcome is adding a polynomial term.

We reproduce the equation from the manuscript here:

Y _ij_ = a + u _i_ + b f(Age) _ij_ + c_1_ Length _ij_ + c_2_ Weight for Height _ij_ + c_3_ TSF _ij_ + c_4_ SSF _ij_ + c_5_ Non-Asian _i_ + e _ij_ where e _ij_ ~ N (0, σ^2^) and u _i_ = N (0, τ^2^) **[1]**

Addition of the quadratic polynomial of Age would imply f(Age) = b_1_ Age + b_2_ Age^2^. However, polynomial regression has several drawbacks (Perperoglou 2019 BMC Med Res Met).

Splines present a statistically reproducible way of modeling non-linearity, without trading off substantive interpretability. The easiest of such models is the linear spline. As described in the manuscript, we model Age as consisting of two knots that join three segments: f(Age) = Age + (Age-9) *I (Age ≥ 9) + (Age-18) * I (Age ≥ 18). Here, Age is associated with the outcome across its range. The term (Age-9) *I (Age ≥ 9) is applicable only from Age 9 months onwards, and (Age-18) * I (Age ≥ 18) is applicable only from Age 18 months onwards. An issue with linear splines is that it involves sharp points (non-smooth) that are non-differentiable.

Natural splines provide us with smooth functions of the exposure-outcome association over the range of the exposure. This is achieved by a transformation of the exposure over its range into a set of ‘basis functions’. Natural splines are specified using two parameters over the range of the exposure – the number of knots and the degree of the polynomial. In our prediction models, we fit natural splines with four degrees of freedom, implying three interior knots at 25^th^, 50^th^ and 75^th^ percentile apart from two boundary knots at minimum and maximum values of the exposure. The degree of the polynomial is usually set to 3, implying cubic polynomials are used since they are visually smooth.

***Prediction interval***

We estimate the uncertainty intervals for validation and test data using two methods: (a) bootstrapping that uses unconditional values for data with previously unobserved levels for estimating the confidence intervals, and (b) interval estimation using conditional modes of random effects that incorporates residual variance and uncertainty from fixed effects for prediction intervals. Both methods ignore uncertainty in variance of grouping factors (random intercepts of individual trajectories in our case). The latter would be wider.
