# Supplementary material for: Anthropometric prediction models of body composition in 3 to 24month old infants: a multicenter international study
Source: Eur J Clin Nutr. 2024 Sep 20;78(11):943–51. doi: 10.1038/s41430-024-01501-0 (PMC11537960; doi:10.1038/s41430-024-01501-0)
Supplement: Supplementary file 10 — Supplementary Note 2 [file 41430_2024_1501_MOESM10_ESM.docx]

# Supplementary Note 2. Comparison of error metrics for quantifying model fit

Different measures of error in model prediction have advantages and drawbacks. We define the error in model prediction (e_i_) as (y_i_ - $\hat{y_{i}})$

RMSE = $\sqrt{\frac{1}{N} \sum_{1}^{N} \left( e_{i} \right)^{2}}$

RMSPE (%)=100. $\sqrt{\frac{1}{N} \sum_{1}^{N} \left( \frac{e_{i}}{y_{i}} \right)^{2}}$

MAE = $\frac{1}{N} \sum_{1}^{N} |e_{i}|$

MAPE (%) = 100. $\frac{1}{N} \sum_{1}^{N} |\frac{e_{i}}{y_{i}}|$

1. RMSE and RMSPE are more sensitive to outliers than MAE and MAPE. The optimization of the former aims to be closer to the mean, while the optimization of the latter would try to be closer to the median.

2. RMSE and MAE are in the units of the outcome variable, while MAPE and RMSPE are percentages calculated relative to the predicted quantity. The former are agnostic to the magnitude of the predicted quantity, while the latter depend on the predicted quantity, such that high errors for lower values of the outcome will impact the latter.
